# Effect of the TiO_2_ Colloidal Size Distribution on the Degradation of Methylene Blue

**DOI:** 10.3390/nano13020302

**Published:** 2023-01-11

**Authors:** So-Yul Kim, Tae-Geol Lee, Seon-Ae Hwangbo, Jong-Ryul Jeong

**Affiliations:** 1 Nanosafety Team, Safety Measurement Institute, Korea Research Institute of Standards and Science (KRISS), 267 Gajeong-ro, Yuseong-gu, Daejeon 34113, Republic of Korea; 2 Department of Materials Science and Engineering, Graduate School of Energy Science and Technology, Chungnam National University, 99 Daehak-ro, Yuseong-gu, Daejeon 34134, Republic of Korea

**Keywords:** TiO_2_, nanoparticle, microparticle, methylene blue, degradation, ultrasonic dispersion, advanced oxidation process

## Abstract

TiO_2_ is the most commonly used photocatalyst in water treatment. The particle size of TiO_2_ is an important factor that significantly influences its activity during photocatalytic degradation. In the presence of liquid, the properties of nanopowders composed of exactly the same product clearly differ according to their aggregation size. In this study, TiO_2_ nanoparticles with a controlled size were fabricated by focused ultrasound dispersion. The high energy generated by this system was used to control the size of TiO_2_ particles in the suspension. The constant high energy released by cavitation enabled the dispersion of the particles without a surfactant. The activities of the prepared TiO_2_ photocatalysts for methylene blue (MB) degradation were then compared. The dye degradation effect of the photocatalyst was as high as 61.7% after 10 min when the size of the powder was controlled in the solution, but it was only as high as 41.0% when the aggregation size was not controlled. Furthermore, when the TiO_2_ concentration exceeded a certain level, the photocatalytic activity of TiO_2_ decreased. Controlling the size of the aggregated photocatalyst particles is, therefore, essential in water-treatment technologies utilizing TiO_2_ photocatalytic properties, and adjusting the TiO_2_ concentration is an important economic factor in this photocatalytic technology. This study contributes to the development of processes for degrading dyes, such as MB, released from wastewater into aquatic environments.

## 1. Introduction

Water pollution due to industrial activities has become a serious environmental problem [1,2]. In particular, the textile and dyeing industries are major sources of water pollution [3]. Among various industrial dyes, methylene blue (MB), an organic cationic dye [4,5], is non-biodegradable, toxic, carcinogenic, and can pose a threat to human health and the environment. Excessive exposure to MB can result in breathing difficulties, digestive disorders, dermatitis, and blindness [5,6,7,8,9,10].

Several studies involving the degradation of MB with advanced oxidation processes (AOPs) have been published [11]. AOPs generate hydroxyl radicals that are highly reactive and cause non-selective reactions. The photocatalysis method is an AOP in which complex molecules, such as dyes, are degraded into low-molecular-weight, non-toxic products, such as H_2_O and CO_2_, using highly reactive substances such as hydroxyl radicals. In such a way, this process can safely remove dyes from wastewater [12,13,14].

TiO_2_ is a typical photocatalytic material used in the photocatalytic degradation of MB [13,14,15,16,17,18,19,20]. When irradiated by ultraviolet (UV) light with a wavelength corresponding to the energy bandgap of TiO_2_, a redox reaction is induced that creates electron–hole pairs, and the electrons are maintained in an excited state. The electrons in the conduction band reduce oxygen to produce peroxide anions, and the holes in the valence band oxidize water molecules to generate hydroxyl radicals. The generated hydroxyl radicals and peroxide anions then degrade MB [12].

However, the particle size of TiO_2_ affects its degradation ability. Decreasing the particle size increases the surface-area-to-volume ratio, which improves its photocatalytic properties [12,15,18]. In a photocatalytic reaction, the contact area between the reactants and the photocatalytic material ultimately affects the degradation performance. Consequently, TiO_2_ nanoparticles provide greater photocatalytic activity than larger particles at the same volume because they have greater surface area per volume [21,22]. Unfortunately, the recombination rate of electron–hole pairs increases with the decrease in the particle size, thus decreasing the efficiency of photocatalytic degradation [23]. Since the photocatalysis property of TiO_2_ is affected by the particle size and its distribution [24,25,26], the TiO_2_ particle size should be optimized to maximize its degradation performance.

Meanwhile, the attractive force between nanoparticles is stronger than that between microparticles owing to the interactions (e.g., van der Waals forces) between the particles. Therefore, nanoparticles are susceptible to agglomeration in solution [27]. Consequently, surfactants are used to maintain the TiO_2_ nanoparticles in a dispersed state, which increases production costs and potentially introduces environmental pollution. Furthermore, dispersion methods such as ball milling, wherein nanoparticles are physically dispersed, contaminate the dispersion solution and disperse particles unevenly as the balls make contact with the particles during the dispersion process [28,29]. Therefore, ultrasonic dispersion has attracted attention as a method for dispersing nanoparticles without direct contact. Several studies have been conducted on how to control agglomeration without direct contact with the dispersed colloid [30,31]. However, in the cases of bath, horn, and cup ultrasonic systems, the energy reaching the colloid is low, and the particles are not uniformly dispersed. Furthermore, controlling the heat generated is not easy [32,33,34,35,36].

In the process of decomposing MB, the concentration of the TiO_2_ photocatalyst in the MB solution affects the photocatalytic activity. A large amount of TiO_2_ does not necessarily provide excellent photocatalyst characteristics. If the TiO_2_ concentration increases above a certain level in the solution, the photocatalytic characteristics decrease. Therefore, the concentration of particles in the solution must also be regulated when optimizing the photocatalytic characteristics, in addition to controlling the size of the aggregated particles.

Recently, an ultrasonication system was reported [37] that relies on non-contact ultrasonic dispersion and can provide excellent dispersion stability to dispersed TiO_2_ nanoparticles without surfactants. In this study, we used this focused ultrasonic dispersion method to disperse TiO_2_ as aggregates of different sizes without a surfactant, and the degradation of MB as a function of the size of TiO_2_ aggregates was evaluated. For simplicity, we hereafter refer to these aggregates, which are irreversible assemblages of primary nanoparticles, as particles.

## 2. Materials and Methods

### 2.1. Materials and Instruments

TiO_2_ Degussa P25 particles with a mean diameter of 25 nm and density of 3.78 g/cm^3^ were purchased from Evonik. An aqueous MB solution (M2661, 0.1%; SAMCHUN Chemicals, Co., Ltd., Pyeongtaek, South Korea) was diluted to prepare the MB solution. A Bio Link crosslinker (BLX, Vilber Lourmat, Collégien, France) was used to irradiate the MB solution during the experiments. In addition, the solvent used in all experiments was deionized water, which was produced using a Direct-Q(R) 3 UV Water Purification System (ZRQSVP3EU, Merck Millipore, Burlington, VT, USA) and had a resistance of 18.2 MΩ·cm.

### 2.2. Dispersion of TiO_2_ Particles in Water

TiO_2_ nanoparticles were dispersed in water without a surfactant and using a high-intensity focused ultrasound device (FS-R01K1, FUST Lab, Daejeon, South Korea) [37,38,39]. First, acoustic energy was focused on the center of a rod passing through an ultrasound device by a cylindrical piezoelectric ceramic. During this time, a large amount of heat was generated, and the temperature was controlled by the energy transfer medium (cooling water). Temperature-controlled ultrasonic systems can operate for longer times than other ultrasonic equipment. The energy was focused until extremely high energy levels were concentrated on the center of the chamber, which dispersed the aggregated TiO_2_ colloids passing through the system. The dispersion stability of the water-dispersed TiO_2_ suspension obtained through this process could be maintained for a long time without a surfactant [37].

The dispersion was performed at 400 kHz and at a power of 150 W. The speed at which the suspension passed through the center of the circular piezoelectric ceramic (PZT) was 5.1 mL/min using the pump (WT600-1F, LONGER, Amersham, UK). When the dispersion conditions were adjusted, a laser particle size analyzer (LA-960S, HORIBA, Kyoto, Japan) was used to measure the size of the dispersed TiO_2_ particles in suspension, which varied according to the dispersion time (3 min, 10 min, 1 h, 4 h, and 24 h). The sizes of the final TiO_2_ colloidal aggregates in the ultrasonicated TiO_2_ suspension (1 wt%) were in the range of 90–4000 nm, depending on the dispersion time. The degree of degradation of MB for each size range was also studied.

### 2.3. Degradation of Methylene Blue

The TiO_2_ suspensions that were ultrasonically dispersed in deionized water at 1 wt% were further diluted in 0.2 g/L and 0.4 g/L of deionized water and remained in suspension. In addition, 20 ppm MB solution was further diluted 50-fold, and the two diluted samples were mixed in a 1:1 weight ratio. Finally, degradation experiments were conducted on the MB solution (10 ppm) with 0.1g/L and 0.2g/L TiO2 concentrations, which was stirred in the dark at 200 rpm for approximately 30 min to enable the TiO_2_ particles to adsorb the MB molecules. After stirring, the MB solution was irradiated by UV-A light (365 nm). Approximately 1.5 mL of the MB solution was collected every 10 min for 60 min, and the absorbance was measured using a UV-visible spectrophotometer (UV-180, Shimadzu, Kyoto, Japan). The TiO_2_ particles were removed from the collected solution using a syringe filter to exclude their influence. The degradation (%) of MB was calculated using Equation (1) [40]:

(1) $$\mathrm{Degradation}\;(\mathrm{absorbance})\;(\%)\;=\;(\frac{A_{0}-A}{A_{0}})\times 100,$$

where *C*_0_ and *C* are the concentrations of MB before and after degradation, respectively, and *A*_0_ and *A* are the absorbances of MB before and after degradation, respectively.

As the concentration and absorbance are proportional according to the Beer–Lambert law, the change in the concentration of MB can be calculated from the degradation (%), which was measured using the optical absorbance of MB, as 664 nm [41].

## 3. Results

### 3.1. Size Distribution of the TiO_2_ Suspension

The mean particle size and the peak value of the particle size distribution of each TiO_2_ sample, i.e., the size range to which the greatest number of particles belong, were measured twice for each sample. Each TiO_2_ sample was dispersed under the same dispersion conditions except for the dispersion time, which was found to affect the particle size distribution and average particle size. The resulting mean and mode particle sizes are shown in Table 1.

The results revealed that without ultrasonic dispersion, the colloids comprised TiO_2_ microparticles (Table 1; sample 1). By controlling the ultrasonic exposure time and output power, dispersed TiO_2_ colloids with mean particle sizes of 2900, 2000, 726, 600, and 90.7 nm were obtained.

**Figure 1:**
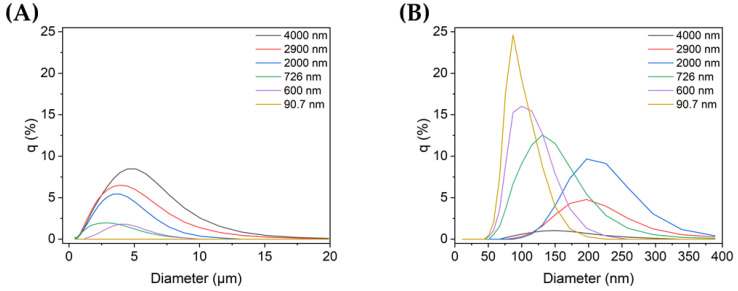
TiO_2_ particle size distribution in non-dispersed and dispersed colloid samples (q (%): the percentage in each histogram channel): distributions of (**A**) microscale and (**B**) nanoscale particles in each sample.

Figure 1A,B show the size distributions of each sample for micro- and nanoscale particles, respectively. The TiO_2_ sample with the smallest particles (90.7 nm) did not contain any microparticles, i.e., all of its particles were on the nanoscale, and its mode size was 81.9 nm. The rest of the samples contained both micro- and nanoparticles. Among them, sample 1 (4000 nm), which was not ultrasonicated, showed the highest peak in the microscale, with a mode size of 4200 nm. As the mean particle size decreased, the distribution at the microscale also decreased, whereas the nanoparticle distribution increased.

### 3.2. Photodegradation

#### 3.2.1. Photocatalytic Effect of TiO_2_ Particles by UV Irradiation

**Figure 2:**
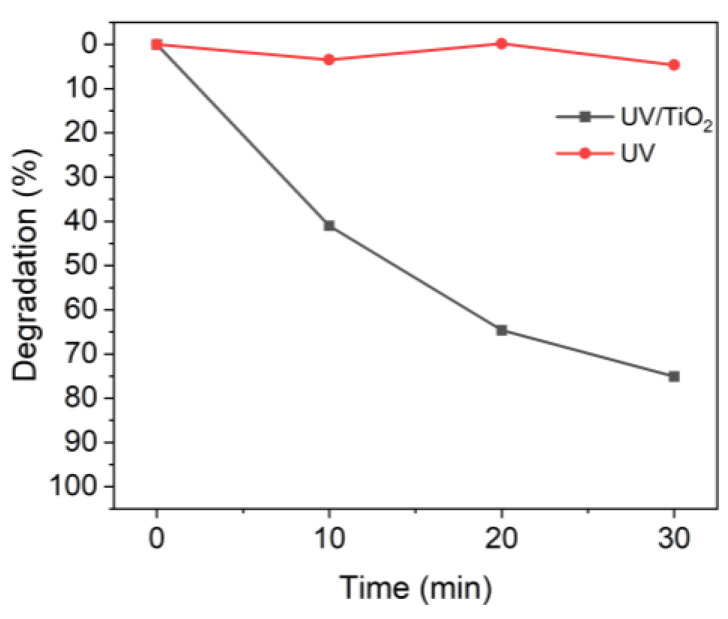
Degradation of MB upon UV irradiation in the presence or absence of TiO_2_.

Figure 2 shows the effect of TiO_2_ on the degradation of MB when irradiated with UV-A light [42]. The TiO_2_ used was not degraded. In the presence of TiO_2_, 10 min of UV irradiation degraded approximately 40% of the MB, whereas almost no degradation occurred in the absence of TiO_2_.

#### 3.2.2. Effect of the TiO_2_ Particle Size Distribution on Photocatalysis

Next, the effect of the TiO_2_ particle size on the degradation of MB was studied. Experiments were conducted using a MB solution with a concentration of 10 ppm after mixing a MB solution (20 ppm) and the TiO_2_ suspensions with different particle sizes (0.2 g/L) in a 1:1 ratio.

**Figure 3:**
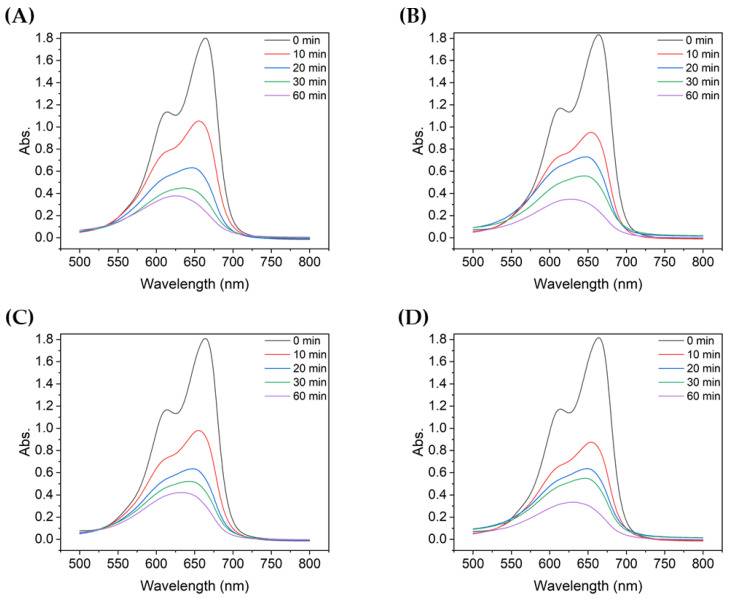

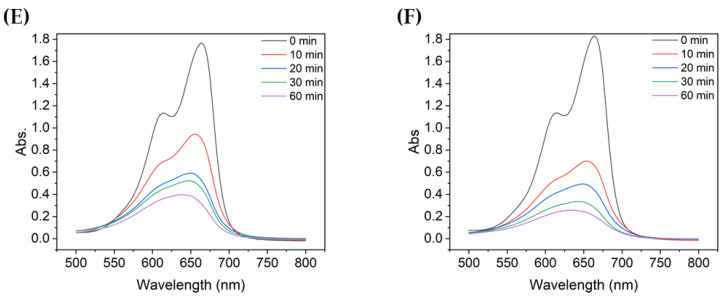
UV-vis absorbance spectra of MB after degradation using TiO_2_ colloids with different mean particle sizes: (**A**) 4000 nm, (**B**) 2900 nm, (**C**) 2000 nm, (**D**) 726 nm, (**E**) 600 nm, and (**F**) 90.7 nm.

**Figure 4:**
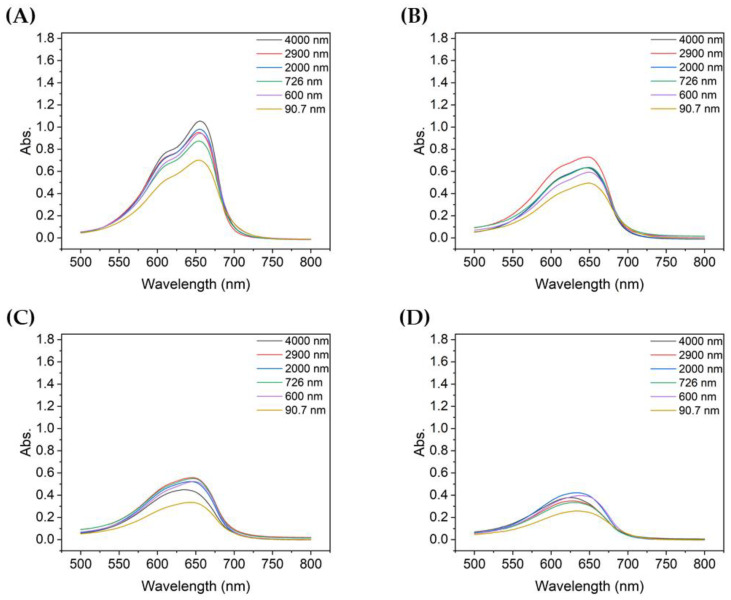
Absorbance spectra of MB after degradation using TiO_2_ colloids for different UV irradiation times: (**A**) 10 min, (**B**) 20 min, (**C**) 30 min, and (**D**) 60 min.

Figure 3 shows the absorbance of MB after degradation with the TiO_2_ suspensions with different particle sizes. For all TiO_2_ samples, the absorbance peak decreased significantly during the first 10 min of UV irradiation. During the first 10 min, the non-ultrasonicated TiO_2_ particles (Figure 3A) showed a decrease in absorbance from approximately 1.8 to 1.05, whereas the more ultrasonicated TiO_2_ particle suspensions showed a decrease in absorbance from ~1.8 to 0.60–0.80. The degree of decrease varied with the particle size. Therefore, even with the same type of powder, the agglomeration size influenced the degradation of dyes such as MB, as clearly shown in Figure 4.

Figure 4 compares the absorbance at each particle size with the same UV irradiation duration. For TiO_2_ of all particle sizes, the absorbance of the decomposed MB gradually decreased as the degradation time increased from 10 to 60 min. Among them, the absorbance of MB decomposed by sample 6, which utilized the most dispersed suspension with a mean particle size of 90.7 nm, was the lowest for all UV irradiation times. In addition, the shorter the degradation time, the greater the difference in absorbance of the decomposed MB solution, depending on the size of TiO_2_ aggregated particles; similarly, the better the dispersion, the lower the peak absorbance.

**Figure 5:**
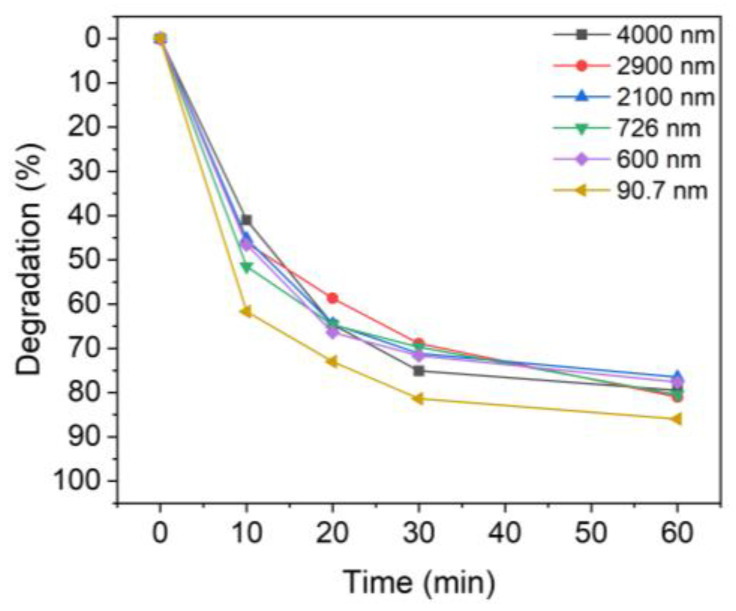
Degradation of MB upon UV irradiation using TiO_2_ colloids with different TiO_2_ particle sizes: 4000 nm, 2900 nm, 2100 nm, 726 nm, 600 nm, and 90.7 nm.

Figure 5 shows the MB photocatalytic degradation results for different TiO_2_ particle sizes. After UV irradiation for 60 min, the colloidal particles with mean sizes of 4000, 2900, 2000, 726, 600, and 90.7 nm degraded 79.5%, 81.0%, 76.5%, 80.5%, 77.6%, and 86.0% of MB. Notably, while the degradation was less than 80% when using micro-sized TiO_2_, the degradation rate with nanoparticles of TiO_2_ was 86%. For the dispersed TiO_2_ solutions, sample 6 (90.7 nm) was slightly more efficient than those with other particle sizes. This difference was more evident when the UV irradiation time was short.

Table 2 shows the degradation of MB after 10 min of UV irradiation time. Evidently, the degree of degradation of MB for each particle size showed a difference of up to ~20% compared to the non-sonicated sample, which degraded only 41.0% of the MB. In the case of the smallest particles (sample 6), about 61.7% of MB was decomposed in 10 min. Therefore, TiO_2_ nanoparticles with better dispersion, i.e., a smaller aggregate size, are advantageous for the degradation of dye solutions such as MB.

#### 3.2.3. Photocatalytic Effect of the Concentration of TiO_2_ Particles

**Figure 6:**
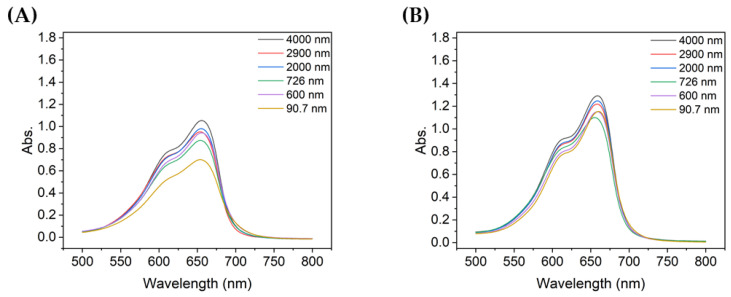
Absorbance spectra of MB after 10 min of degradation using colloids with different TiO_2_ concentrations: (**A**) 0.1 g/L and (**B**) 0.2 g/L.

**Figure 7:**
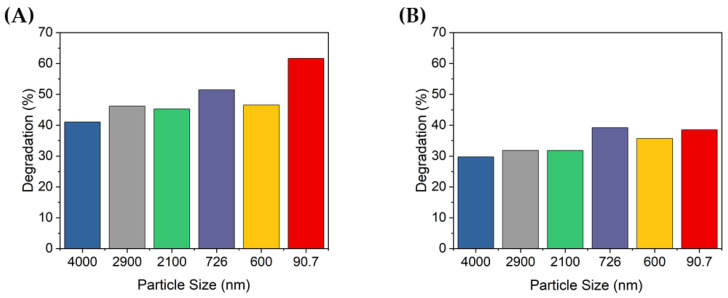
Degradation of MB after 10 min UV irradiation using TiO_2_ colloids with different TiO_2_ concentrations: (**A**) 0.1 g/L and (**B**) 0.2 g/L.

The effect of the TiO_2_ concentration on the degradation of MB was also studied. These experiments were carried out in the same way as the previous ones except for the TiO_2_ concentration. The difference in the MB degradation between TiO_2_ concentrations of 0.1 and 0.2 g/L is shown in Figure 6 and Figure 7.

Figure 6A,B show the absorbance of the MB degraded for 10 min when the concentration of TiO_2_ was 0.1 g/L or 0.2 g/L, respectively, with the same MB concentration solution. When the concentration of TiO_2_ was 0.1 g/L, the peak absorbance was lowest with the 90.7 nm TiO_2_ particles, whereas at 0.2 g/L, the 726 nm TiO_2_ particles showed the lowest absorbance. When the concentration of particles of TiO_2_ was 0.1 g/L, the absorbance peaks were notably different depending on the size of the aggregated particles, but this difference was less significant when the concentration was 0.2 g/L. Furthermore, the absorbance peak of degraded MB, which was relatively less degraded at 0.1 g/L, was lower than that at 0.2 g/L, thereby confirming that the TiO_2_ concentration affects the degradation of MB.

Figure 7 shows the percentage of degradation according to the peak absorbance shown in Figure 6. At 10 min of UV irradiation, approximately 41–61.7% and 29.7–39.2% of the MB was degraded at concentrations of 0.1 and 0.2 g/L, respectively. At 0.2 g/L, MB was degraded up to 39.2% by the 726 nm TiO_2_ particles, but this was less degradation than that of the non-ultrasonicated TiO_2_ particles when the TiO_2_ concentration was 0.1 g/L (i.e., 41%). When the concentration of TiO_2_ was 0.1 g/L, the degradation degree of the MB solution was significantly different by up to 20%, depending on the size of the dispersed particles. In contrast, at a concentration of 0.2 g/L, there was no significant difference in the degradation degree of MB according to the dispersed TiO_2_ particle size. Furthermore, when the concentration of TiO_2_ increased, the degradation performance decreased. Therefore, the concentration of TiO_2_ was confirmed to affect the degradation of dyes such as MB; specifically, if the concentration is higher than a certain level, the photocatalytic activity may decrease.

## 4. Discussion

In this study, the agglomeration of particles was controlled only by ultrasonic dispersion without a surfactant when TiO_2_ powder (P25), which is commonly used in research and industry, was aggregated in a liquid solution such as water. Consequently, the mitigation of MB according to the size of the dispersed TiO_2_ particles could be studied. Particles were dispersed not by a general ultrasonic device but by a focused ultrasonication system in which energy is concentrated on the center by a piezoelectric ceramic with a cylindrical structure, which use high energy to disperse the particles. Particles were dispersed by 150 W of power at a frequency of approximately 400 kHz, and the particle size changed depending on the dispersion time. Specifically, the longer the dispersion time, the smaller and more evenly dispersed the particles, and the longer the dispersion state was maintained.

As mentioned above, TiO_2_ samples with different agglomeration sizes showed different photocatalytic activities for MB. When the TiO_2_/UV reaction was applied to a MB solution (10 ppm), different levels of degradation were observed for each size, especially when the UV irradiation time was short. At a TiO_2_ concentration of 0.1 g/L, TiO_2_ with the smallest particle size of 90.7 nm degraded MB by about 61.7% based on a UV irradiation time of 10 min, but TiO_2_ with aggregates (4 µm) that were not dispersed by ultrasonication only degraded about 41%. Therefore, although the same type of powder was used, the degradation rate was varied by more than 20% as a result of us controlling the size of aggregates in the solution.

Furthermore, when the concentration of MB was the same, its degradation depended on the concentration of TiO_2_. When 0.1 g/L of TiO_2_ was present in the solution, the degradation performance was superior to that with 0.2 g/L. In the presence of TiO_2_ with a size of 90.7 nm at a concentration of 0.1 g/L, the degradation was 61.7%, but at 0.2 g/L, the degradation was only approximately 38.6%. Thus, when the UV irradiation time was the same for TiO_2_ of the same size, the degradation was ~1.5 times different depending on the TiO_2_ concentration. Other sizes of TiO_2_ showed similar differences depending on the concentration.

## 5. Conclusions

This study examined the effects of the size and concentration of TiO_2_ powder aggregates in aqueous solution on the degradation of MB. A focused ultrasonic system was used to control the agglomeration of TiO_2_ powders. This system was useful for dispersing aggregated particles without the use of surfactants.

The TiO_2_ agglomerations were controlled at different sizes, and the degradation performance of ultrasonicated particles of different sizes was up to ~20% higher than those of particles whose agglomeration size was not controlled. Accordingly, the photocatalytic properties of TiO_2_ for MB degradation were more efficiently activated when their particle size and distribution were well controlled. In addition, when the concentration of the photocatalyst particles in the dye solution exceeded a certain level, their photocatalytic activity decreased. This finding indicates that an optimum concentration leading to well-activated photocatalytic properties exists.

Future studies could examine the degradation of dye solutions such as MB in a variety of ways, including photocatalytic methods, and research on more efficient dye degradation should be conducted. This study was part of a research project aimed at removing the chromaticity of dye wastewater released into the aquatic environment, which is expected to improve the aquatic environment.

## Figures and Tables

**Table 1:** Mean and mode TiO_2_ particle sizes for each sample.

Sample	Sonication Time (min)	Mean (nm)	Mode (nm)
1	0	4000	4200
2	3	2900	3700
3	10	2100	187.1
4	60	726	122.8
5	240	600	94
6	1440	90.7	81.9

**Table 2:** Degradation (%) at a UV irradiation time of 10 min.

Sample	Mean Size (nm)	Degradation (%)
1	4000	41.0
2	2900	46.2
3	2100	45.3
4	726	51.5
5	600	46.6
6	90.7	61.7

## Data Availability

The datasets used and analyzed in the current study are available from the corresponding author on reasonable request.
